# Through the Smoke: An In-Depth Review on Cigarette Smoking and Its Impact on Ocular Health

**DOI:** 10.7759/cureus.47779

**Published:** 2023-10-27

**Authors:** Aryan Kulkarni, Shashank Banait

**Affiliations:** 1 Department of Ophthalmology, Jawaharlal Nehru Medical College, Datta Meghe Institute of Higher Education and Research, Wardha, IND

**Keywords:** age-related macular degeneration (armd), macular degeneration, ocular health, smoking cessation, dry eye syndrome, cataracts, glaucoma, ocular diseases, cigarette smoking

## Abstract

Smoking is a widespread and pervasive habit, impacting health across various care settings, including acute care, subacute care, home-based care, and long-term care. Smoking is a serious global public health concern that has been related to many chronic diseases. However, the effect of smoking on eye disorders has been less studied. Cigarette smoke contains a complex mixture of harmful constituents, including nicotine and toxic chemicals, which permeate the bloodstream, affecting ocular tissues. The oxidative stress and inflammation induced by smoking are central to its detrimental effects on ocular health. Age-related macular degeneration (AMD), a leading cause of vision loss, exhibits a strong association with smoking. Research consistently demonstrates that smokers face a heightened risk of both early and advanced AMD. Cataracts, another prevalent ocular condition, develop earlier and progress more rapidly in smokers. The oxidative stress on the lens and reduced antioxidants among smokers contribute to the increased severity of cataracts. Moreover, the health of the eyes may be compromised by smoking-related chemicals that reduce blood flow and/or hasten thrombus formation in ocular capillaries thus increasing the chance of acquiring glaucoma, cataracts, AMD, and Graves' eye disease. Beyond individual health concerns, the societal implications of smoking on ocular health are substantial, including increased healthcare costs and diminished quality of life for affected individuals. Understanding the underlying mechanisms can provide insights into potential therapeutic interventions for preventing and managing smoking-related ocular damage. Given the global prevalence of smoking, raising awareness about the ocular risks associated with smoking is crucial for promoting eye health. The review underscores the urgent need for comprehensive anti-smoking initiatives and smoking cessation programs to alleviate the burden of ocular diseases associated with smoking.

## Introduction and background

Smoking is a direct cause of lung cancer and is linked to malignancies of the bladder, breast, and colon. While most cancers are caused by the interactions of genetic and environmental factors, smoking is a direct cause of lung cancer [1]. Smoking has damaging effects on the eyes as well [2]. Numerous common and serious eye conditions, including Graves' ophthalmopathy, age-related macular degeneration (AMD), glaucoma, and cataracts, have been proven to be at risk [3]. Many of these illnesses result in permanent blindness. Additionally, there is proof that smoking has a dose-response impact on eye morbidity. Glaucoma, which is a major global cause of permanent vision loss, is one of the ocular illnesses associated with cigarette smoking that has been the subject of the most research. The progressive damage to the optic nerve that characterizes the complex multifactorial disease known as glaucoma is frequently linked to increased intraocular pressure [4]. Numerous studies have shown a correlation between smoking and an elevated risk of acquiring the condition, making smoking a modifiable risk factor for glaucoma. Although the exact mechanisms underlying this link are not yet fully understood, it is thought that the harmful substances found in cigarette smoke, such as free radicals and substances that cause oxidative stress, may play a role in the onset and progression of glaucoma. Smoking has also been connected to vascular dysfunction and poor blood flow regulation, which can worsen glaucoma-related eye damage [5]. Smoking has also been associated with AMD, a primary cause of permanent visual loss in older people. The core region of the retina, the macula, which is crucial for clear and detailed vision, is impacted by AMD. Numerous studies have repeatedly shown that smokers are more likely to acquire AMD than non-smokers. Smoking is thought to have negative consequences for AMD that are mediated by oxidative stress, inflammation, and vascular damage. The delicate equilibrium of the ocular environment can be upset by the harmful compounds included in cigarette smoke, which can result in AMD-related pathological alterations. Smoking has also been shown to accelerate the advancement of AMD in those who already have it, raising the chance of profound visual impairment. Cataracts, a clouding of the lens of the eye that causes hazy vision, are another ocular disorder that is closely linked to smoking [6]. Epidemiological studies have repeatedly shown that smokers have a higher chance of developing cataracts. Although the precise mechanisms by which smoking causes cataracts to occur are yet unknown, oxidative stress and damage to lens proteins are thought to be major contributors. Smoke from cigarettes contains harmful substances and reactive oxygen species that can cause oxidative damage and cause lens opacity. Smoking also compromises the lens' capacity to combat oxidative stress and maintain transparency because it has been linked to lower antioxidant levels in the ocular tissues. Dry eye syndrome, a common ocular condition marked by inadequate tear production or subpar tear quality, has also been linked to smoking [7]. According to studies, smokers are more susceptible to dry eye symptoms and have less stable tear films than non-smokers. In conclusion, cigarette smoking represents a significant modifiable risk factor for a variety of ocular diseases, including glaucoma, AMD, cataracts, and dry eye syndrome [8]. The precise mechanisms underlying this association are not yet fully understood, but they may involve increased tear evaporation, inflammation, and alterations in the composition of tear film components due to the toxic substances in cigarette smoke. Smoking has several harmful consequences for eye health, including oxidative stress, inflammation, vascular dysfunction, and other processes. To effectively promote smoking cessation, increase awareness of the ocular dangers of smoking, and lessen the burden of smoking-related ocular illnesses, it is crucial to understand the relationship between smoking and ocular diseases. Healthcare professionals, legislators, and the general public can work together to protect ocular health and stop vision loss brought on by smoking-related ocular illnesses by treating smoking as a modifiable risk factor [9].

## Review

Methodology

**Figure 1:**
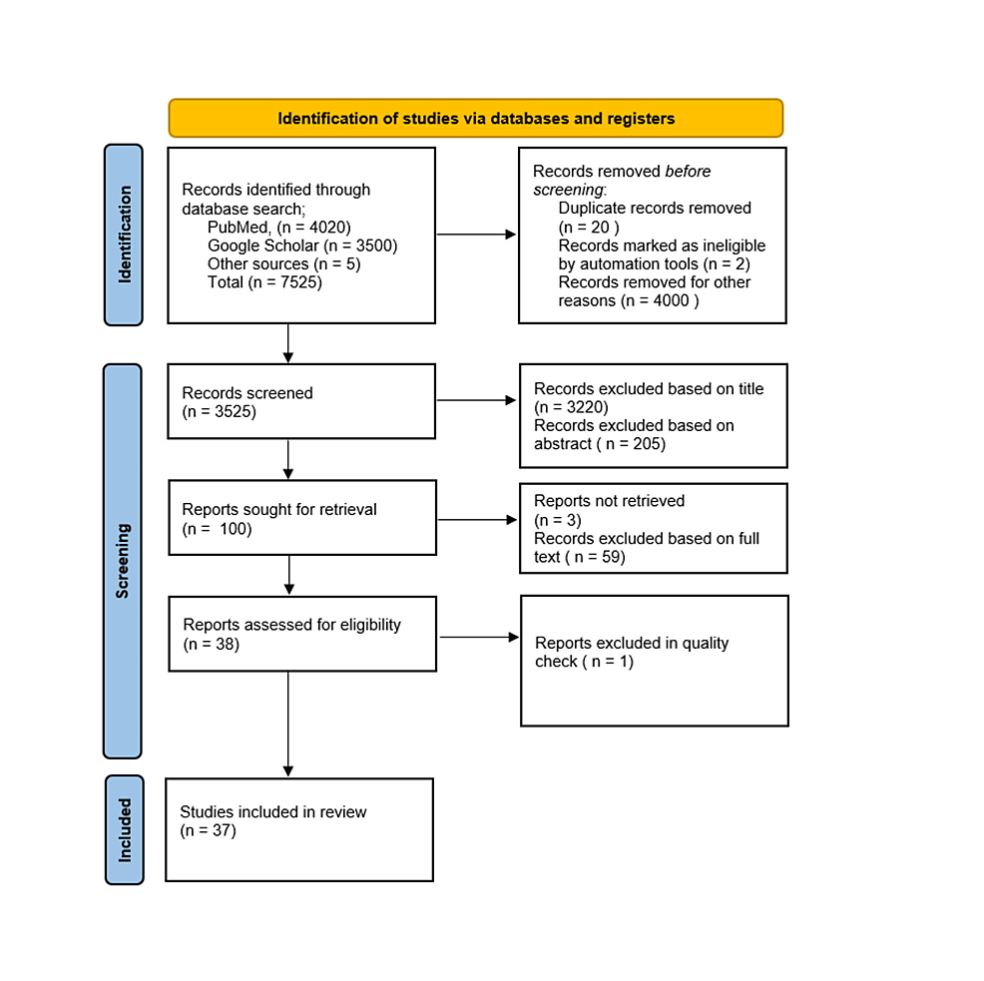
PRISMA flow diagram Adapted from the Preferred Reporting Items for Systematic Reviews and Meta-Analyses (PRISMA) flow diagram 2020.

We undertook a systematic search through PubMed, Google Scholar, and CENTRAL in June 2023 using keywords such as "smoking and ocular health" or "cigarettes and ocular diseases" or "management of smoking and ocular health" ((smoking and ocular health[Title/Abstract]) OR ("smoking and ocular health "[MeSH Terms]), (cigarettes and ocular diseases [Title/Abstract])) OR (cigarettes and ocular diseases [MeSH Terms]), (("management of smoking and ocular health"[Title/Abstract]) OR ("management of smoking and ocular health [MeSH terms])). The selection of the studies (Figure 1) depended on the following inclusion criteria: (1) smoking and ocular health; (2) management of smoking and ocular health; (3) cigarettes and ocular health; and (4) English language. The following were the exclusion criteria: (1) case study; (2) effect of other smoke on ocular health; (3) animal studies; (4) bench research; (5) not empirical study (e.g., theory or opinion articles); and (6) non-English language research. Related articles over a period of the last 10 years were searched, which included free full text, reviews, book articles, website reports, and online published reports. About 67 articles were obtained. After screening for duplicates, suitability, inclusion, and exclusion criteria, a total of 67 articles were shortlisted. A total of 37 articles were included in the final review. Figure 1 shows the Preferred Reporting Items for Systematic Reviews and Meta-Analyses (PRISMA) flow diagram for searched articles.

Discussion

Glaucoma

Glaucoma is an optic neuropathy for which high intraocular pressure is a key risk factor. Glaucoma is a frequent and paramount cause of blindness that affects most countries, although smoking and intraocular pressure do not substantially connect. A new population-based investigation of 3752 non-glaucoma adults aged 40 to 84 years in the Caribbean identified a minor association between smoking and higher intraocular pressure (P = 0.05), but greater correlations with other risk factors like diabetes and hypertension. Since smoking raises intraocular pressure, a substantial risk factor for open-angle glaucoma, it may be a secondary cause of the disorder [10]. A case-control analysis indicated that smokers are more likely than non-smokers to get glaucoma. A different population-based analysis with 4926 individuals, however, revealed that glaucoma prevalence did not alter in connection to cigarette smoking status. Environmental factors such as diet, alcohol usage, and smoking are known to have a considerable influence on glaucoma, just as they do on macular degeneration. Smoking is one environmental element that may alter the optic nerve and raise the risk of glaucoma. An experimental study reveals that the relationship between smoking and glaucoma is biologically based. Smoking instantly boosts intraocular pressure by 5 mmHg, according to research on the dynamics of aqueous humor [10]. The episcleral venous pressure could increase as a consequence of selective vasoconstriction, resulting in an obstruction of the outflow tract [10]. Increased intraocular pressure is directly connected with more severe glaucomatous damage in primary open-angle glaucoma [11]. Numerous defects in the anterior chamber of the eyes may cause the intraocular pressure to rise. Environmental factors, which also include age, family history, smoking, and alcohol consumption, account for 10% of the variance in intraocular pressure. Despite the assumption that smoking may be an avoidable risk factor for glaucoma, studies on how smoking affects intraocular pressure have yielded inconsistent findings, making it impossible to establish a clear causal relationship between the two. Further study is necessary on this link, which may be sensitive to the interplay with other factors [12].

Age-Related Macular Degeneration (AMD)

The presence of either soft drusen or any sort of drusen, if linked to alterations in the retinal pigment epithelium or an increase in retinal pigmentation in the macular area, is referred to as early AMD. It is a significant contributor to blindness in the West and the main factor in severe vision impairment in the elderly [13]. According to estimations, 1.5% of Caucasians have advanced AMD. The incidence rises with age. According to a recent study, among 355 older people over 40 years who were randomly picked for a visual assessment, 19% had both AMD and cataracts. The key reason causing blindness in AMD is the degeneration of the retinal pigment epithelium. The photoreceptors' damage is one of the secondary reasons. Whether or not there are difficulties with vascular invasion, AMD frequently manifests as a restricted version of the disease that kills the important rods and cones. Even though only 10% of AMD patients have the exudative form of the illness, this sort of disease is responsible for more than 85% of the legal blindness connected with the ailment [14]. Age, smoking, diet, sun exposure, and hypercholesterolemia are all risk factors for AMD. Research on the association between smoking and the beginning of AMD has been undertaken in epidemiological, case-control, and surveillance settings. Exudative AMD is more prevalent among smokers than non-smokers. Environmental risk factors for progressive AMD were studied by the United States Eye Disease Case-Control Study Group. A total of 421 individuals with neovascular AMD who were older than 55 years were included in the study, along with 615 controls who were age- and sex-matched. Smokers demonstrated a much greater prevalence of AMD than "never smokers" compared to current and former smokers, despite accounting for other risk variables such as blood levels of carotenoids and cholesterol. Both a cross-sectional Beaver Dam Eye sample of persons aged 43 to 86 years and a Japanese sample of respondents aged 50 to 69 years generated comparable pan-ethnic results. There are particular smoking practices that are more likely to be dangerous than others. An increased prevalence of AMD among smokers in Japan has been connected with deep inhalation, smoking without a filter, beginning to smoke before the age of 20 years, and smoking for more than 40 years. The risk of developing AMD is modified by smoking in a dose-dependent way [15]. A prospective study enrolling 157 male physicians in the US indicated that smokers who consume more than 20 cigarettes per day have a two- to three-times increased likelihood of having AMD with vision loss. Even years after they had quit smoking, former smokers retained a heightened risk. There was a 1.76 relative risk of having AMD and vision loss among former heavy smokers who had quit smoking less than 20 years prior to the trial's commencement. A much greater prevalence of AMD with vision loss was reported among former heavy smokers who had stopped smoking 20 years or more prior to the research [16]. Theoretically, inadequate intracellular biomolecule clearance and breakdown contribute to aberrant cellular metabolism, which in turn causes retinal pigment epithelium degeneration in AMD. This aberrant metabolism leads to a buildup of undesirable metabolic byproducts within the epithelium. According to one concept, smoking may encourage the creation of new subretinal capillaries, which leads to the choriocapillaris forming a tubular capillary network. There are additional arguments for the relationship between smoking and AMD as well. Second, smoking may result in atherosclerosis and hypoxia in the choroidal vasculature, which may lead to disciform macular degeneration. The retinal pigment epithelium has been penetrated by choroidal neovascularization. Smoking increases total and low-density lipoprotein (LDL) cholesterol levels, platelet adhesiveness, and fibrinogen concentrations while lowering plasma high-density lipoprotein (HDL) cholesterol levels. Thirdly, oxidants generated during phagocytic cell activity or those present in cigarette smoke may increase retinal oxidative stress. The peroxidation of polyunsaturated fatty acids is increased by rising oxidative stress. These results stimulate microinfarction, ischemia, and hypoxia in the macula as well as increased carboxyhemoglobin levels stimulate, worsen macula aging, and obstruct choroidal blood flow. See Table 1 for the age and sex distribution sample [17].

**Table 1:** Age and sex distribution sample Adapted from [17].

	Smokers		Non-smokers		P
	Mean	SD	Mean	SD	
Age (years)	38	14.8	34	15.3	0.23
Sex					
Male	37	93%	22	57.4%	0.00007
Female	1	3%	16	42.4%	0.00003

Smoking is commonly recognized as an independent risk factor for AMD (the exudative variety in particular) with a dose-response link, with more positive than negative data often supporting this view [18].

Graves' Eye Disease

Graves' disease (exophthalmic goiter), like the majority of autoimmune disorders, has a complex etiology. The exact cause of Graves' disease is unknown, but genetic and environmental factors interact to create the disease's pathogenetic mechanism. Many Graves' disease patients also have subclinical eye disorders. Smoking has been extensively researched for Graves' ophthalmopathy. In smokers with Graves' ophthalmopathy, elevated serum levels of thyroxine and thyroid-stimulating hormone (TSH) receptor autoantibodies are not always detected. However, chronic smokers often have somewhat lower TSH levels and higher thyroglobulin levels. Smokers often have more goiters [19]. Epidemiological studies support the harmful effects of smoking on Graves' ophthalmopathy. Smokers are more likely than non-smokers to develop Graves' ophthalmopathy. Smoking frequency also correlates with the severity of the ocular disorder; those with Graves' disease who smoke more frequently have more severe eye conditions. Smoking is an independent risk factor for Graves' disease, but its association with smoking in Graves' ophthalmopathy is even stronger and statistically more significant. In a case-control study involving 400 subjects, smoking significantly increased the risk for Graves' ophthalmopathy with hyperthyroidism after adjusting for confounders like sex and age [20]. Smoking also elevated the probability of having Graves' sickness. Smoking raises the likelihood of the illness in all ethnic groups, but among patients with active, moderate-to-severe Graves' ophthalmopathy, smokers had lower results with orbital irradiation treatment than non-smokers.

On the question of the relationship between smoking and Graves' sickness, numerous ideas have been put forth [21]. One investigation indicated that compared to European smokers, who had a noticeably increased likelihood of having Graves' ophthalmopathy, Asian smokers, the majority of whom were of Indian and Pakistani heritage, had a considerably lower risk. According to one theory, smoking may have an influence on the immune system. Compared to non-smokers, smokers demonstrated reduced levels of immunosuppression and T-suppressor lymphocyte activity. Smoking may reduce immunological surveillance, which may limit the capacity of spontaneously created T-helper cell clones to control antigens present in the thyroid or orbit [22]. In addition, stimulating the synthesis of a thyroid autoantigen that interacts with ocular muscle and changes its suppleness may be employed as a therapy for the immunological consequences of smoking. The etiology of autoimmune thyroid disease is largely impacted by this decreased control. Indirect proof of the immune system's influence of smoking comes from the anthracene chemical 3-methylcholanthrene, which induces autoimmune thyroiditis in rats. Superoxide improved cell proliferation in cultures of fibroblasts from retro-ocular connective tissue obtained from Graves' ophthalmopathy patients in a dose-dependent manner. Orbital edema is induced by the proliferation of retro-ocular fibroblasts and the release of glycosaminoglycans into the extracellular matrix. Graves' ophthalmopathy is distinguished by this volume increase. Interleukin 1 (IL-1)'s pro-inflammatory and fibrogenic properties also contribute to volume growth [23]. Due to their much weaker reactivity to orbital irradiation, patients with Graves' ophthalmopathy have significantly lower blood levels of the naturally occurring IL-1 receptor antagonist, which suppresses IL-1's effects. Smoking is connected with Graves' ophthalmopathy, according to epidemiological and experimental examinations of eye disorders. These patients are avid cigarette smokers. Furthermore, it has been found that antibodies to the heat shock protein are present in both Graves' disease patients and healthy, non-smoking adults. Smoking promotes eye diseases. Smoking raises the risk of Graves' ophthalmopathy by affecting the clinical course and treatment outcome. Patients, especially those at risk, should be persuaded to stop smoking even if the specific mechanism creating the negative consequences is currently unknown and there may be several mechanisms at work [24].

Cataract

The term "cataract" describes how the lens has become opaque. There are three different anatomical forms of cataracts: nuclear, cortical, and posterior subcapsular. Nuclear sclerosis, which is more pronounced in elderly people, is what causes nuclear cataracts. The central location has a considerable impact on vision [25]. The cortical layers of the lens that are furthest from the visual axis are affected by cortical cataracts. "Onion skin" cataracts near the posterior pole of the lens are referred to as posterior subcapsular cataracts. They are connected to metabolic conditions such as homocystinuria, galactosemia, hypoparathyroidism, and diabetes mellitus. With a best-corrected visual acuity of 3/60 or below, cataracts are the main cause of blindness in the world and are responsible for sight loss in more than half of the 23 million individuals [26]. Even if surgery is the only accessible and effective treatment, figuring out the risk factors can help create preventative measures. There is a dose-response relationship between the cumulative amount of smoking and the risk of developing a nuclear cataract. Heavy smokers are more at risk than other groups [27]. Case-controlled, cross-sectional, and prospective studies have comprehensively explored the epidemiological connection between smoking and cataracts. However, the influence of smoking on non-nuclear opacity is still up for discussion. When undergoing their first eye checkup, people who smoke 20 or more cigarettes per day are considerably more likely than non-smokers to acquire nuclear opacity, according to a 30-year research study. Additionally, compared to people who only smoke 20 or fewer cigarettes per day, those who smoke more than 20 cigarettes per day have a considerably greater risk. Nuclear cataracts were more typically connected with pipe smoking than with cigarette smoking. These findings might be explained by differences in smoking patterns rather than a real difference between smoking a pipe and smoking cigarettes. Users of pipes are less likely to inhale than users of cigarettes, and pipes presumably create more side-stream smoke. This additional smoke may cause serious lung harm. Cataracts may arise in many different ways and are brought on by a variety of factors [28]. Smoking is just one of numerous recognized or suspected risk factors for cataracts, along with advanced age, trauma, chronic intraocular inflammation, UV radiation, diabetes mellitus, hypoparathyroidism, extended corticosteroid therapy, and a high body mass index. Smoking may indirectly raise the lens' oxidative stress by lowering the intake of nutrients such as ascorbic acid and nicotinamide, which have antioxidant properties. Additionally, chemicals like cadmium or isocyanate that are found in cigarette smoke or its metabolites may damage lenses both structurally and directly [29]. In organ-grown rat lens tissue, condensation products of wood smoke have been observed to accumulate and contribute to morphological issues like hyperplasia, hypertrophy, and the multilayering of epithelial cells. Similar histological abnormalities and higher calcium levels were identified in the lenses that had been taken out of rats that had been exposed to cigarette smoke for a total of two hours each day for 60 days at a time. These findings give clear evidence that in vivo exposure to cigarette smoke may damage lenses [30]. To definitively establish that smoking promotes cataract development, a longer, more complete assessment that is unaffected by other variables is required [31].

Dry Eye Disease

The multifactorial condition known as dry eye is characterized by changes to the ocular surface that weaken the tear film and cause eye itching, redness, light sensitivity, blurred vision, and/or other symptoms and/or discomforts. According to estimates, 5% to 35% of people worldwide experience dry eye at varying ages. More than 3.2 million females and 1.6 million men over the age of 50 years in the United States experienced moderate or severe dry eye [32]. Numerous causes have been proposed for dry eye, including chronic ocular surface inflammation, decreased corneal and conjunctival sensitivity, decreased tear production and/or stability, and epithelial damage. Smoking is a well-known risk factor for numerous chronic diseases, including those that damage the eyes, as was previously discussed [33]. The health of the eyes may be compromised by smoking-related chemicals that reduce blood flow and/or hasten thrombus formation in ocular capillaries thus increasing the chance of acquiring glaucoma, cataracts, AMD, and optic neuritis. Although this is not yet confirmed, smoking may make you more likely to have dry eyes in general [34]. Smoking may alter the stability of tears as well as the corneal and conjunctival sensitivity. Smoking has also been associated with ocular discomforts like burning and a foreign body sense. However, the Schirmer score findings did not demonstrate a notable shift. At the 2007 International Dry Eye Workshop, the tear breakup time (TBUT) of 10 seconds and the 5-mm Schirmer score administered without anesthesia were suggested as the diagnostic criteria for dry eyes. Older age and female sex were two of the dry eye risk factors that were the most inconsistent. It had been established that sex hormones affected the ocular surface's homeostasis [35]. See Table 2 for a comparison of dry eye parameters between smokers and non-smokers [36].

**Table 2:** Comparison of dry eye parameters between smokers and non-smokers Adapted from [36].

Variable	Smokers		Non-smokers		P
	Mean	SD	Mean	SD	
	Schirmer’s II test	22.25	12.63	28.84		10.23	0.0125
Tear meniscus height	0.22	0.04	0.35	0.11	<0.00010
Tear breakup time	9.68	3.95	12.81	1.92	<0.00010

Along with age and sex, the environment may impact the likelihood of dry eye. For instance, it has been found that the risk of acquiring dry eyes is raised for both office employees who use visual display terminals (VDTs) and glaucoma patients who use anti-glaucoma medicines. The findings of the meta-analysis that only included studies employing samples from the general population found no indication of a relationship between current smoking and dry eyes. The studies we included in our core meta-analysis were related to populations that were exposed to additional dry eye risk factors, including the use of VDTs, or the studies that were linked to specialist populations, like hospital-based populations, which may explain the discrepant findings [37].

## Conclusions

In conclusion, smoking's harmful effects on eye health are widely known and demand serious consideration. Several visual disorders, including glaucoma, AMD, cataracts, and dry eye syndrome, have been reliably linked to cigarette smoking. Smoking harms ocular tissues in a variety of ways, including oxidative stress, inflammation, vascular dysfunction, and other intricate processes. Smoking has been linked to glaucoma, which is characterized by gradual damage to the optic nerve. According to studies, smoking is a modifiable risk factor for glaucoma that carries a higher risk for smokers. Promoting smoking cessation as a primary preventative measure is critical since smoking cessation interventions have a significant impact on lowering glaucoma risk and disease progression. Smoking has also been connected to AMD, a primary cause of irreversible visual loss in older people. AMD is more likely to occur in smokers than in non-smokers, but quitting smoking can lower both the risk and severity of the condition. Healthcare providers can aid in lowering the prevalence of AMD by promoting smoking cessation and increasing public knowledge of the dangers smoking poses to the eyes. The opacity of the lens, which is a hallmark of cataracts, has long been linked to smoking. The damaging substances in cigarette smoke cause oxidative stress and lens protein degradation, which aid in cataract development. Quitting smoking has been found to reduce the likelihood of developing cataracts and postpone their onset, demonstrating its potential as a potent preventive intervention. Smoking has also been related to dry eye syndrome, which is characterized by inadequate tear production or poor tear quality. Smoking cessation plays a key role in treating this widespread ocular illness by reducing the symptoms of dry eyes and enhancing tear film function. In conclusion, the overwhelming body of research shows that smoking is bad for your eyes. Smoking cessation programs are essential for reducing risk, slowing the advancement of different ophthalmic disorders, and improving their outcomes. To protect eye health and lessen the burden of smoking-related ocular disorders, healthcare practitioners, policymakers, and the general public should prioritize quitting smoking. We can work to improve ocular health outcomes and contribute to a healthy future for people all around the world by increasing awareness, offering help for quitting, and putting effective tobacco control measures in place.
